# Antiviral Activity of Beebread, Bee-Collected Pollen and Artificially Fermented Follen against Influenza A Virus

**DOI:** 10.3390/foods12101978

**Published:** 2023-05-13

**Authors:** Tilemachos G. Dimitriou, Nikos Asoutis Didaras, Christina Barda, Dimitra Skopeliti, Katerina Kontogianni, Katerina Karatasou, Helen Skaltsa, Dimitris Mossialos

**Affiliations:** 1 Laboratory of Microbial Biotechnology, Molecular Bacteriology-Virology, Department of Biochemistry and Biotechnology, School of Health Sciences, University of Thessaly, 42500 Larissa, Greece; tidimitr@uth.gr (T.G.D.); didasout@uth.gr (N.A.D.); dskopeliti@uth.gr (D.S.); katerinakontojohn@gmail.com (K.K.); 2 Department of Pharmacognosy and Chemistry of Natural Products, Faculty of Pharmacy, School of Health Sciences, National and Kapodistrian, University of Athens, Zografou, 15771 Athens, Greece; cbarda@pharm.uoa.gr (C.B.); skaltsa@pharm.uoa.gr (H.S.); 3 Apicultural Centre of Larissa, Federation of Greek Beekeepers Associations, 41500 Larissa, Greece; info@omse.gr

**Keywords:** honey bee products, bee bread, bee-collected pollen, chemical profile, antiviral activity, influenza A virus

## Abstract

Bee-collected pollen (BCP) and the naturally fermented BCP product known as bee bread (BB) are functional foods renowned for their nutritious, antioxidant, antibacterial and other therapeutic properties. This is the first study employed to assess the antiviral activity of BCP and BB against influenza A virus (IAV) H1N1 along with their proteinaceous, aqueous and *n*-butanol fractions. Additionally, artificially fermented BCP has been evaluated against IAV (H1N1). Antiviral activity was assessed in vitro by comparative real-time PCR assay. IC_50_ values ranged from 0.022 to 10.04 mg/mL, and Selectivity Index (SI) values ranged from 1.06 to 338.64. Artificially fermented BCP samples AF5 and AF17 demonstrated higher SI values than unfermented BCP, and proteinaceous fractions demonstrated the highest SI values. The chemical profile of BCP and BB samples, analyzed using NMR and LC-MS, revealed the presence of specialized metabolites that may contribute toward the antiviral activity. Overall, the significant anti-IAV activity of BB and BCP harvested in Thessaly (Greece) could be attributed to chemical composition (especially undiscovered yet proteinaceous compounds) and possibly to microbiome metabolism. Further research regarding the antiviral properties of BCP and BB will elucidate the mode of action and could lead to new treatments against IAV or other viral diseases.

## 1. Introduction

Bee-collected pollen (BCP) and beebread (BB) are honey bee products renowned for high nutritional value, as they provide proteins, essential amino acids, sugars, fatty acids (ω-3 and ω-6 included), vitamins and important macro- and microelements. BCP and BB contain a plethora of bioactive compounds commonly present in functional foods, such as prebiotics, probiotics, fibers, lignans, carotenoids, triterpenes, and organic acids [1,2,3]. Furthermore, they are rich in polyphenols and demonstrate significant antioxidant activity [4]. BB is actually produced by BCP fermentation and is then stored and preserved in the hive for a long time. BCP fermentation is catalyzed by bee enzymes and inoculated microorganisms (bacteria, molds and yeasts), leading to a more nutritious product compared to BCP [5]. Few studies have demonstrated the antimicrobial activity exerted by BB against major bacterial pathogens and fungi, which might be attributed to phytochemicals (polyphenols, terpenes, phytosterols) as well as to antimicrobial compounds presumably produced by the BB microbiome [6,7,8,9]. Regarding antiviral activity, some honey bee products, in particular propolis and honey, have been adequately studied (recently reviewed in [10,11]). However, data presenting antiviral activity of BB and BCP are scarce. Recently, Didaras et al. have demonstrated for the first time the antiviral activity exerted by BCP and BB against enterovirus D68 [12].

Furthermore, Lee et al. [13] have characterized the neuraminidase inhibitory activity of specific BCP compounds (six flavonoids and one alkaloid) present in Korean *Papaver rhoeas* L. against influenza A strains. Influenza A virus (IAV) infects various avian and mammalian hosts. Spread of seasonal IAV strains is considered a serious public health issue, especially among the oldest and immunocompromised. The IAV genome comprises eight gene segments of single-stranded, negative-sense RNA. The IAV virions have spike-like projections at the outer surface (resembling in that sense SARS-CoV-2), which are composed of two surface glycoproteins: hemagglutinin (HA) and neuraminidase (NA). The existing high antigenic variability within surface glycoproteins classifies the members of IAV into 18 HA and 11 NA subtypes [14].

Vaccines and antiviral drugs (M2 ion channel inhibitors, neuraminidase and polymerase inhibitors) are used for the prevention and treatment of influenza viruses. However, viral resistance to approved antiviral medicines and vaccines has exacerbated the need to discover new antiviral compounds to combat influenza [15,16].

The aim of this study was to analyze the chemical profile of BCP and BB samples harvested in Greece as well as to assess in vitro the antiviral activity exerted by unprocessed whole bee products, derived fractions and artificially fermented BCP against IAV (H1N1).

## 2. Materials and Methods

### 2.1. Samples and Sample Preparation

In this study, two bee-collected pollen (BCP) samples and two beebread (BB) samples were further processed, as described below, giving in total 19 tested samples (Table 1).

Every single BB and BCP sample was firstly dissolved in Minimum Essential Medium (MEM) with Earle’s Balanced Salts cell culture medium (MEM, LM-E1144, Biosera, France) for one hour (1 h) at room temperature, centrifuged for 10 min at 10,000× *g*, and then, the supernatant was filtered through a Branchia 0.22 μm syringe filter (Labbox Labware, S.L., Barcelona, Spain). Filtered BB and BCP suspensions were serially diluted and used for the MMT and antiviral assays described below.

In a previous study conducted in our lab on the biological properties of Greek BB [7], palynological analysis of 18 BB samples was performed. Samples 1-BB and 4-BB correspond to samples 1 and 4, whose detailed botanical composition has been presented in the Supplementary Material of the aforementioned publication.

Palynological analysis has been also published for sample 6P-BCP, which was tested in a previous study conducted in our lab on the in vitro antiviral activity of Greek BB and BCP against Enterovirus D68 [12]. For sample AF1, no palynological analysis was performed, but along with the sample, the beekeeper provided information on the major families of blooming plants in the area during BCP collection. According to this information, the dominant plant families were Brassicaceae, Ranunculaceae, Papaveraceae and Cistaceae. Palynological analysis was conducted by CheMa laboratories (Korinthos, Greece) as previously described [7].

#### 2.1.1. Isolation and Identification of Microorganisms Applied in BCP Fermentation

Bacteria and yeasts were isolated from a polyfloral fresh BB sample harvested in Thessaly, Greece (October 2021). Bacteria were grown on diverse solid media by spreading 100 μL of different BB dilutions (10^−1^, 10^−2^ and 10^−3^) in sterile saline (0.9 % *w*/*v*). Six plates of each dilution were prepared: three of them were incubated at 37 °C and the rest at 30 °C at least for 5 days. The following media were used: plate count agar (PCA) (Neogen Heywood UK), *Bacillus cereus* medium (LAB M, Bury, UK), containing 1.5% bacteriological agar and polymyxin B (LAB M, Bury, UK), and Man Rogosa and Sharpe (MRS) agar (Neogen, Heywood, UK) plates incubated anaerobically in an AnaeroJar AG25 using the AnaeroGen Atmosphere Generation system (Oxoid, Basingstoke, UK) at 30 °C for approximately 5 days. In the case that several morphologically identical colonies appeared on a plate, at least three of them were picked up. Single colonies were used for further subcultures and testing. Yeasts were grown on plates containing 45% glucose, 5% fructose, 0.5% yeast extract (YE), 0.5% NaCl and 1.5% agar in order to selectively isolate osmophilic yeasts adapted in honey and pollen [17]. They were further subcultured on Potato Dextrose Agar (PDA) (LAB M, Bury, UK) as described above for bacteria. Obtained bacterial and yeast isolates were stored as glycerol stocks in Nutrient broth (LAB M, Bury, UK) supplemented with 0.5% YE, 0.5% glucose and 20% *v/v* glycerol at −80 °C, until further analysis.

Bacteria and yeasts were identified by 16S rRNA gene and Internal Transcribed Spacer (ITS) region sequencing, respectively. A single colony was used for genomic DNA extraction with the ExtractMe Genomic DNA Kit (Blirt, Gdansk, Poland).

For 16S rRNA gene amplification universal primers 27F (5′-AGAGTTTGATCMTGGCTCAG-3′) and 1492R (5′-GGTTACCTTGTTACGACTT-3′) (Eurofins Genomics, Germany) were used to amplify the 16S rRNA gene by PCR, as described in a previous study conducted in our lab [18].

For ITS amplification, ITS1 (5′-TCCGTAGGTGAACCTGCGG-3′) and ITS4 (5′-TCCTCCGCTTATTGATATGC-3′) (Eurofins Genomics) were used. The reaction mixture contained: 2 U FastGene Taq DNA Polymerase, 1X PCR buffer A, MgCl_2_ 0.5 mM, 25 pmol of each primer, 1 mM dNTPs, 3 μL DNA template, and deionized sterile water to a final volume of 50 μL. A thermal cycler Primus 25 was used in the following PCR conditions: initialization at 95 °C for 3 min, followed by 40 cycles of denaturation at 95 °C for 30 s, annealing at 55 °C for 30 s, and elongation at 72 °C for 30 s. A final elongation step at 72 °C for 2 min was added.

Amplicons were purified using the NucleoSpin Gel and PCR clean-up kit (Macherey-Nagel, Düren, Germany) and then sequenced via the Sanger dideoxy termination method by CeMIA SA (Larissa, Greece). Chromas version 2.6.6 software (Technelysium Pty Ltd., South Brisbane, Australia) was applied to check the quality of the obtained sequences. Sequences were assembled into a single sequence via MEGA X version 10.1.6 software [19] and Gene Runner version 6.5 software (www.generunner.net, accessed on 11 October 2022) and were subjected to a BlastN (Megablast) search in NCBI GenBank to identify sequences with the highest similarity.

#### 2.1.2. Starter and Additional Cultures

Starter and additional cultures regarding BCP solid state fermentation were prepared as follows: Overnight bacterial and yeast cultures were grown on Nutrient Broth supplemented with 0.5% YE and 0.5% glucose (for bacteria) and Potato Dextrose broth (for yeast) at 37 °C. Cultures were centrifuged at 4000× *g* for 5 min, and the pellet was resuspended in saline. Cultures of *Apilactobacillus kunkeei* were grown in Nutrient Broth supplemented with 0.5% YE and 0.5% glucose for 48 h at 30 °C, bacterial cells were centrifuged at 4000× *g* for 5 min, and the pellet was resuspended in saline. BCP was grounded using a sterile mortar and pestle.

Multifloral BCP (AF1, Table 1) was used as substrate throughout all fermentations. BCP quantity, inoculation strains and fermentation conditions of each fermented sample are described below.

AF4 sample was fermented as follows: 11.5 g of AF1 inoculated with 10^8^ CFUs of *Z. siamensis* were resuspended in 2.3 mL sterile saline. The sample was mixed with sterile spatula and incubated for 8 days at 33 °C.

AF5 sample was fermented as follows: 11.5 g of AF1 inoculated with 10^8^ CFUs of *A. kunkeei* were resuspended in 2.3 mL saline. The sample was mixed with sterile spatula and incubated for 29 days at 33 °C.

AF17 sample was fermented in three stages:

1. A total of 11.5 g of AF1 inoculated with 10^8^ CFUs of *Z. siamensis* was resuspended in 2.3 mL sterile saline. The sample was mixed with sterile spatula and incubated for 24 h at 33 °C.
2. A total of 2.5 g of the above product (A) inoculated with 10^8^ CFUs of *A. kunkeei* was resuspended in 250 μL sterile saline. The sample was mixed with sterile spatula and incubated for 3 days at 33 °C.
3. A total of 11.5 g of AF1 was added to B, mixed and inoculated with 10^8^ CFUs of *Bacillus licheniformis*, 10^8^ CFUs of *Bacillus* sp. and 10^8^ CFUs of *Bacillus subtilis* resuspended in 1 mL sterile saline. The sample was mixed with sterile spatula and incubated for 24 h at 33 °C, then pressed using sterile pestle and incubated for 28 days at 33 °C. *Bacillus* strains were selected after screening for their ability to produce diverse enzymes (unpublished data).

At the end of the incubation period, 1 g of each sample was diluted in 10 mL of distilled water, and pH values were measured using a JENWAY pH meter.

#### 2.1.3. Proteinaceous Fractions of BCP, BB, Fermented BCP and Proteinase-K Treatment

In order to prepare the proteinaceous fractions, 500 mg of each initial sample was firstly dissolved in sterile deionized water (ddH_2_O) up to a final volume of 1.5 mL. The samples remained for 1 h at room temperature with occasional stirring. Then, the samples were centrifuged for 10 min at 10,000× *g*, and the supernatant aqueous fraction was collected. Subsequently, solid ammonium sulfate (NH_4_)_2_SO_4_ (AnalaR, BDH Chemicals Ltd., Poole, England) was gradually added up to a final concentration of 60% *w/v*. Samples were stirred gently and left for 20 min at room temperature for equilibration. Then, they were incubated for 10 min at −20 °C and centrifuged for 30 min at 11,000× *g*. The supernatant was discarded, and the pellet was resuspended in 1 mL sterile ddH_2_O [20,21]. Furthermore, proteinaceous fractions were treated with 150 μg/mL proteinase-K (Blirt, Poland) for 24 h at 37 °C at 210 rpm, and then, the fractions were tested for antiviral activity.

#### 2.1.4. Ethyl Acetate, n-Boutanol and Aqueous Fractions

To obtain the ethyl acetate, *n*-butanol and aqueous fractions, BCP and BB samples (≈500 mg each) were dispersed in water (10 mL × 3), vortexed for approx. 1 min and then incubated overnight at room temperature. After centrifugation for 10 min at 4000× *g*, the supernatant aqueous fraction was collected and filtered, and the samples were further subjected to liquid–liquid extraction with ethyl acetate (10 mL × 3) (Merck, Darmstadt, Germany) and *n*-butanol (10 mL × 3) (Merck, Darmstadt, Germany), successively, to extract the non-polar constituents (lipids; ethyl acetate fraction; 1-BB-Et, 4-BB-Et, 6P-BCP-Et), the medium-polar components (polyphenols, sugars; *n*-butanol fraction; 1-BB-Bu, 4-BB-Bu, 6P-BCP-Bu), respectively, and the remaining aqueous fractions with polar constituents (sugars and proteins; aqueous fraction; 4-BB-H, 6P-BCP-H). All obtained fractions were concentrated to dryness under vacuum (20 °C) and placed in activated desiccators with P_2_O_5_ until their weights were stabilized. Samples were analyzed by GC-MS (fractions 1-BB-Et, 4-BB-Et, 6P-BCP-Et), LC-MS (fractions 1-BB-Bu, 4-BB-Bu and 6P-BCP-Bu) and NMR (fractions 1-BB, 4-BB, 6P-BCP, 1-BB-Et, 4-BB-Et, 6P-BCP-Et, 1-BB-Bu, 4-BB-Bu and 6P-BCP-Bu).

### 2.2. Chemical Analysis of BCP and BB Fractions

#### 2.2.1. Nuclear Magnetic Resonance (NMR) Spectroscopy

The equipment and methodology employed for the NMR analysis have been previously described by Tsami et al. [22]. During the NMR analysis, a metabolomic strategy was employed to continuously monitor and trace all extracts and obtained subfractions. This allowed for the characterization of their chemical profiles. Additionally, the 1D and 2D NMR spectra of the n-butanol residues (fractions 1-BB-Bu, 4-BB-Bu, and 6P-BCP-Bu) were measured. The metabolites were identified using NMR experiments, and their chemical shifts and coupling constant values were compared with those of standards and in conjunction with LC-MS data. All candidate structures were then compared to previously published literature (including Reaxys, Scifinder, and an in-house natural compound database).

#### 2.2.2. Liquid Chromatography High-Resolution Quadrupole Time-of-Flight Mass Spectrometry (LC-Q-TOF-MS/MS)

LC-Q-TOF-MS/MS analyses were performed on the n-butanol residues of each sample (1-BB, 4-BB, and 6P-BCP) using the equipment and methodology described in our previous publication (Tsami et al. [22]). In order to obtain the chemical composition, the analysis was conducted in both positive and negative ionization modes. The metabolites were characterized using their mass spectra (their precursor ion and comparison of the fragmentation patterns) by comparing with previously described molecules in the literature using databases such as Reaxys, Scifinder, Pubmed, NIST, mzCloud, and MassBank. The identified compounds were subsequently listed based on their retention times in the total ion chromatogram (TIC).

#### 2.2.3. Gas Chromatography–Mass Spectrometry (GC-MS) Analysis

GC-MS analyses were performed on the ethyl acetate residues (1-BB-Et, 4-BB-Et and 6P-BCP-Et), and the equipment and methodology utilized for the GC-MS analyses were adopted from our previous work as described by Tsami et al. [22].

### 2.3. Cytotoxicity and Antiviral Assays

#### 2.3.1. Cytotoxicity Assay

The MTT (3-(4,5-dimethylthiazol-2-yl)-2,5-diphenyltetrazolium bromide) colorimetric assay [23] was used in order to assess the cytotoxicity of samples against Ma-din-Darby canine kidney (MDCK) cells (Vircell, Granada, Spain), and the protocol used was described before [12].

#### 2.3.2. Influenza A Virus and Cell Culture

A clinical influenza A H1N1 strain isolated and characterized by standard laboratory methods, kindly provided by Dr. Nikolaos Siafakas (ATTIKON General University Hospital of Athens), was used throughout this study. A cell culture assay was implemented in order to assess the antiviral activity of BB and BCP samples against IAV H1N1. MDCK cells in MEM supplemented with 2% fetal bovine serum were seeded in 96-well plates and incubated for 24 h at 37 °C to form a monolayer. Then, the medium was removed, and 200 μL of each sample dilution in MEM medium containing 100 μL IAV H1N1 virus and 1 μg/mL TPCK trypsin (Sigma-Aldrich, St. Louis, MO, USA) were added on the 96-well plate in triplicate. The final concentrations of tested BB and BCP samples ranged from 16 to 0.0625 mg/mL (two-fold dilutions). The plate was incubated at 37 °C and examined daily for development of Cytopathic Effect (CPE).

Wells containing only MDCK cells were used in triplicate as a negative control (cell control), the monolayer of which remained intact throughout the whole experiment. As a positive control, wells containing MDCK cells and H1N1 were used in triplicate (virus control). By the time that 100% CPE appeared in the virus control, the assay ended, and the 96-well plates were stored at −80 °C until further analysis.

Two separate sets of experiments were performed. In the first one, tested samples were incubated for one hour with uninfected MDCK cells before inoculating the IAV H1N1. In the second set of experiments, IAV H1N1was incubated in the presence of tested samples for one hour, and then, MDCK cells were infected.

#### 2.3.3. RNA Extraction and cDNA Synthesis

Each sample was tested in triplicate and pooled in a 1.5 mL tube. RNA extraction was performed using 100 μL of each tube and the chaotropic agent guanidine thiocyanate according to Casas et al. [24]. Finally, the pellet was dried and dissolved in 100 μL of sterile double-distilled, DNase-RNase-free water (DEMO S.A, Athens, Greece).

Since the viral genome of H1N1 virus consists of single-stranded, negative-sense RNA segments, a Reverse Transcription assay was performed, using M30F2/08 primer targeting the M gene segment 5′-ATGAGYCTTYTAACCGAGGTCGAAACG-3′ (Eurofins Genomics, Germany) and FastGene Scriptase II (Nippon Genetics, Japan) according to manufacturer protocol. The synthesized cDNA was stored at −20 °C until further use.

#### 2.3.4. Real-Time PCR Assay

The antiviral activity of samples was assessed by a comparative Real-Time PCR assay and was based on our previous study [12]. The relative concentration of viral titer of different samples compared to the viral titer of virus control was calculated targeting the M gene segment M30F2/08 5′-ATGAGYCTTYTAACCGAGGTCGAAACG-3′/M264R3/08 5′-TGGACAAANCGTCTACGCTGCAG-3′ [25], and the rest of the protocol remained the same.

#### 2.3.5. CC_50_, IC_50_ and SI Calculation

Cytotoxicity concentration 50% (CC_50_) by definition is the concentration of a substance that will kill half the cells in an uninfected cell culture [26]. Sample cell toxicity was calculated using the MTT assay [23].

The half maximal inhibition concentration (IC_50_) is a measure of the potency of a substance to inhibit a specific biological or biochemical function [26]. In our study, IC_50_ was implemented to measure the antiviral potency of BB and BCP samples and the corresponding fractions against H1N1. More specifically, IC_50_ value expressed the concentration of a given sample that corresponds to a 50% decrease in the viral titer and that is calculated via the Real-Time PCR data.

Finally, Selectivity Index (SI) is a ratio that takes into account both cytotoxicity and antiviral activity, and it is calculated from a given CC_50_ value divided by the corresponding IC_50_ value (CC_50_/IC_50_) [27].

## 3. Results and Discussion

### 3.1. Experimental Design

The aim of this study was to assess the antiviral properties exerted by BB and BCP and corresponding fractions against IAV H1N1 virus as well as to elucidate a putative mode of action. Therefore, two distinct sets of experiments were performed. In the first set, we incubated tested samples for one hour with uninfected MDCK cells before inoculating the IAV H1N1. The antiviral activity observed under these conditions would imply that compounds present in the tested samples could inhibit viral infection. In the second set of experiments, we incubated IAV H1N1 in the presence of tested samples for one hour, and then, the MDCK cells were infected. Antiviral activity was observed only in the second set of experiments, indicating that BB, BCP and their proteinaceous fractions exert virucidal activity (see further results in Table 2).

In order to investigate whether the BB microbiome might contribute to antiviral activity, the AF1 BCP sample was inoculated with yeast and bacterial strains isolated from BB, thus simulating solid-state fermentation, which naturally occurs in the hive. After fermentation, the samples AF4, AF5 and AF17 demonstrated lower pH values compared with unfermented AF1 samples (sample pH values AF1: 4.33, AF4: 3.81, AF5: 3.9, AF17: 4.06). The BB microbiome enriches BCP with enzymes, amino acids, vitamins, phenolic compounds and possibly antimicrobial compounds [5,9]. Fermentation of BCP is known to increase the bioavailability of both nutrients and bioactive compounds in BCP [28,29,30]. Honeybees inoculate BCP with lactic acid bacteria, including *Apilactobacillus kunkeei*, and lactic acid fermentation takes place at the initial steps of BCP conversion to BB [6]. Yeasts are present in flower pollen, BCP and BB and actively take part in the fermentation of BCP [28,31]. Osmotolerant yeasts such as *Zygosaccharomyces siamensis* are able to survive in an environment with low water activity, such as honey and BB [32]. Recently, Poyraz et al. artificially fermented BCP using a starter culture, combining strains of *Lactobacillus kunkeei* and two yeasts, *Starmeralla magnolia* MP-2 and *Zygosaccharomyces siamensis* MP-14, to compare it with commercially available BB. They demonstrated that artificially fermented BCP had similar or enhanced qualities in terms of microbial stability and bioavailability of nutrients [33]. Furthermore, a plethora of *Bacillus* strains are present in BB [34], producing cellulases, hemicellulases, amylases and proteinases that breach the hard outer layer of pollen grains and increase the bioavailability of bioactive compounds [35]. For this reason, *A. kunkeei*, *Z. siamensis* and additionally *B. licheniformis*, *B.* sp. and *B. subtilis* strains isolated from our BB samples were implemented in artificial BCP fermentation. To the best of our knowledge, this is the first time that *Bacillus* strains are used alone or in combination with other microorganisms as a starter culture for BCP fermentation.

### 3.2. Cytotoxicity Levels of Tested Samples

Employment of the MTT assay determined the toxicity levels of all tested samples on the MDCK cells. As shown in Table 2, toxicity levels expressed as CC_50_ values ranged from 0.47 to >64 mg/mL, indicating that tested BB and BCP samples might exert cytotoxicity to MDCK cells in relatively low concentrations. In a study by Watanabe et al., toxicity of honey on MDCK cells ranged roughly from 80 to 83 mg/mL [36]. Our data indicate that BCP and BB exhibit higher cytotoxicity on MDCK cells than honey. On the contrary, the proteinaceous fractions of BCP and BB demonstrated lower toxicity compared to the samples from which they were derived, presumably due to lower polyphenol concentration in these samples. Moreover, all three *n*-butanol fractions demonstrated the lowest toxicity on MDCK cells among all tested samples.

### 3.3. Real-Time PCR and Antiviral Activity

A comparative real-time PCR assay was performed to monitor the viral genome copy number in the presence of the tested samples. We postulated that the antiviral activity exerted by the tested samples should lead to a reduced IAV H1N1 genome copy number compared to the positive control (only virus) after co-inoculation of the virus and samples.

Employment of the comparative real-time PCR assay demonstrated a decreasing copy number of IAV H1N1 when the virus is co-inoculated with the tested samples in the cell culture. These data suggest that all BB and BCP samples exert antiviral activity in a concentration-dependent way. The antiviral activity was expressed as IC_50_ values (Table 2) ranging from 0.022 to 10.04 mg/mL.

The *n*-butanol and aqueous extracts did not exert any antiviral effect, with the exception of sample 1-BB-Bu, which demonstrated an IC_50_ value of 2.91 mg/mL. Chemically, sample 1-BB-Bu was differentiated by the presence of quercetin (a known antiviral compound), specific flavonoid glycosides and di-glycosides, as well as by isoflavone derivatives (known antiviral compounds) according to the LC-MS analysis (see below in Table 3). Moreover, the NMR profiling identified predominant characteristic flavonoids (bearing a B-ring without any substituent) such as pinocembrin, pinobanksin and chrysin (see Appendix A).

The proteinaceous fraction of all samples (BB, fermented BCP and BCP) exhibited lower IC_50_ and higher CC_50_ values compared to the corresponding values of the samples from which they were derived. Proteinase-K treatment completely abolished the antiviral activity, thus confirming the proteinaceous nature of the antiviral agent(s) present in these samples.

The antiviral activity was rather variable among samples, which might be attributed to different chemical profiles (Table 3), which in turn depend on the botanical origin and the fermentation process of BB. Wide variations both in composition and concentration of constituents in BCP and BB could be attributed to pre-digestion of pollen grains during BCP fermentation to BB. It has been reported that vitamins, amino acids and other nutrients or metabolites such as phenolic acids might be produced by the BB microbiome, presumably including antimicrobial compounds [5,9,29,35]. In order to test the hypothesis that BB contains antiviral compounds produced by the microbiome, we inoculated BCP with microorganisms isolated and identified from the BB. The product of the solid-state fermentation that took place (designated fermented BCP) was further tested for its antiviral properties (samples AF4, AF5, AF17). AF5 and AF17 samples demonstrated lower IC_50_ values (roughly 1.6 times) than the BCP sample they derived from. The AF4 sample demonstrated a higher IC_50_ value (roughly 2.5 times) than the BCP sample that it derived from. These findings indicate that antiviral activity might be inhibited at least in the case of AF4 by yeast metabolism under different fermentation conditions, compared to *L. kunkei* or the mixed microbial inoculum (AF5 and AF17 samples, respectively).

There are noticeable changes in IC_50_ values between AF and AF-P samples. AF1 and AF1-P IC_50_ values are very similar, 3.91 and 3.14 mg/mL, respectively. However, a noticeable reduction (roughly 3.9 times) between AF4 and AF4-P IC_50_ values (10.04 and 2.71 mg/mL, respectively) was recorded. The most significant reduction (roughly 5.3 times) was observed between AF17 and AF17-P IC_50_ values, from 2.47 to 0.46 mg/mL, respectively. These findings imply a different chemical nature of antiviral substances (proteinaceous versus non-proteinaceous) that might be produced during fermentation. The elevation of CC_50_ values in all AF-P samples compared to AF samples indicated that non-proteinaceous compounds might exert cytotoxicity on MDCK cells (Table 2).

### 3.4. Selectivity Index Values

Selectivity Index (SI) values were calculated for all samples except for those extracts that did not exert any antiviral activity. The higher the SI value, the more effective and safer a substance would be. All samples that exerted antiviral activity demonstrated an SI value higher than 1, meaning that viral inhibition is observed in concentrations that are not toxic for MDCK cells. Seven samples (46%) demonstrated SI values higher than 20, meaning that the IC_50_ concentration is more than 20 times lower than the correspondent CC_50_ concentration, an indication of a potent antiviral effect [27]. As shown in Table 2, SI values are highly variable, ranging from 1.06 to 338.64. Artificially fermented BCP samples AF5 and AF17 demonstrated higher SI values than the raw BCP (AF1), indicating that fermentation could enhance antiviral activity presumably through the release of antiviral phytochemicals present in pollen or due to yet unknown antiviral compounds produced by microorganisms implicated in fermentation.

Finally, proteinaceous fractions in all cases demonstrated higher SI values compared to the samples from which they were derived, and in the case of BB, SI values were impressively high, thus confirming the presence of proteinaceous compounds in BB and exerting high antiviral activity against the influenza virus in combination with low cytotoxicity.

### 3.5. Chemical Analysis of BCP and BB Samples

The aim of this study was not only to assess the in vitro activity of BCP and BB against IAV H1N1 but to elucidate the chemical profile of those underinvestigated honey bee products.

Selected BCP and BB samples are apparently multi-component mixtures of primary and secondary metabolites. They consist of low-molecular-weight components within a mass range of 50–1500 Da as well as unknown polysaccharides and proteins of high molecular weight. Elucidation of this chemical complexity is a rather challenging task for current analytical techniques. In order to successfully identify specific compounds, fractionation of samples based on physicochemical properties prior to subsequent analysis steps is essential. Following fractionation, diverse approaches, including chromatographic methods (LC-MS and GC-MS) and direct spectroscopic methods (NMR), facilitate further chemical analysis [22,37].

In this study, BCP and BB samples were initially characterized by NMR implementation. The ^1^H-NMR spectra clearly revealed their chemical complexity (see Appendix A). Therefore, the samples were further subjected to liquid–liquid extraction, a commonly applied method regarding the complete recovery of constituents. The selected fractionation method involves the partitioning of samples into three main subfractions: (a) The ethyl acetate residue where the lipids are recovered, which is important to be performed before any further analysis. Of note, such residues are not considered bioactive; thus, 1-BB-Et, 4-BB-Et and 6P-BCP-Et fractions were not assessed regarding antiviral activity. (b) *n*-butanol residue, where polar components are extracted, such as polyphenols and sugars. These fractions constitute the phenolic content of BCP and BB samples. Τhus, 1-BB-Bu, 4-BB-Bu and 6P-BCP-Bu were further analyzed by LC-MS and NMR techniques. (c) Lastly, the remaining aqueous fractions contained high-polarity components, including sugars and proteins. Consequently, the complete chemical fingerprints of the initial samples result from the combined analysis of the above fractions.

#### 3.5.1. LC-MS Analysis

LC-MS analysis revealed the presence of more than 45 compounds, including carbohydrates, amino acids, and polyphenols. In Table 3, individual components are listed according to the retention time (*t*_R_) followed by their molecular formulas and the proposed chemical entities. The molecular formulas were established based on high-precision quasi-molecular ions such as [M−H]^−^, [M+H]^+^ or [M+HCOO]^−^, with a mass error of 5.0 ppm, and all data were interpreted and compared with spectra available in the literature and in combination with the obtained NMR data (see Appendix A). Based on these results, the main constituents were confirmed to be carbohydrates and phenolic derivatives. More specifically, the LC-MS results revealed that the samples contain various carbohydrates including mono- and di-saccharides (C_6_H_12_O_6_ *m*/*z* 203.0529 [M+Na]^+^, C_12_H_22_O_10_ *m*/*z* 349.1118 [M+Na]^+^ and C_12_H_22_O_11_ *m*/*z* 365.1056 [M+Na]^+^), amino acids (C_4_H_9_NO_4_ *m*/*z* 135.0542 [M+H]^+^, C_6_H_13_NO_2_ *m*/*z* 132.1018 [M+H]^+^), nuclueosides (C_9_H_12_N_2_O_6_ *m*/*z* 243.0620 [M−H]^−^), and a variety of polyphenols including flavonoid glycosides in the form of aglycons (C_15_H_10_O_7_ *m*/*z* 301.0352 [M−H]^−^, C_15_H_12_O_4_ *m*/*z* 255.0662 [M−H]^−^, C_16_H_12_O_7_ *m*/*z* 315.0511 [M−H]^−^, C_15_H_10_O_6_ *m*/*z* 285.0405 [M−H]^−^, C_15_H_10_O_4_ *m*/*z* 253.0506 [M−H]^−^, C_16_H_12_O_7_ *m*/*z* 315.0508 [M−H]^−^), their mono-/di-/tri- glycosides (e.g., C_21_H_20_O_12_ *m*/*z* 463.0946 [M−H]^−^, C_27_H_30_O_17_ *m*/*z* 625.141 [M−H]^−^, C_33_H_40_O_20_, *m*/*z* 755.2041 [M−H]^−^, respectively), as well as some simple phenolic derivatives (C_9_H_8_O_2_ *m*/*z* 149.0241 [M+H]^+^, C_9_H_8_O_4_ *m*/*z* 179.0350 [M−H]^−^, C_10_H_10_O_4_ *m*/*z* 195.0649 [M+H]^+^). The chemical evaluation was in agreement with previously published data on bee-derived products [8].

#### 3.5.2. NMR and GC-MS Characterization of 1-BB, 4-BB and 6P-BCP Fractions

**Figure A1:**
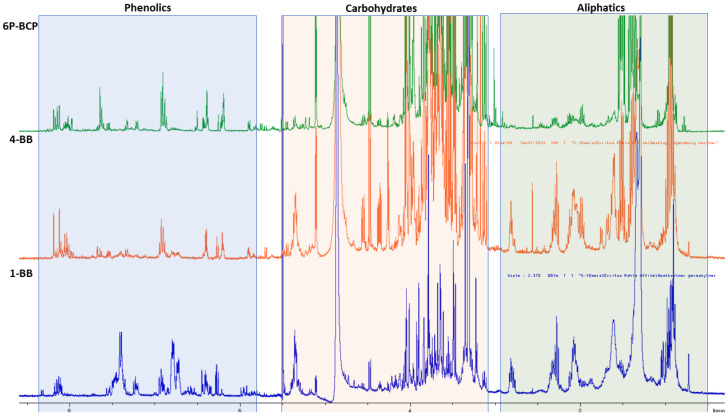
^1^H NMR comparison of the *n*-butanol residues of 1-ΒΒ (blue), 4-ΒΒ (orange), and 6P-BCP (green) in CD_3_OD.

Visual inspection of the NMR spectra revealed the presence of different classes of metabolites as previously discussed in the LC-MS analysis. Phenolic derivatives were detected in all samples, with many common flavonoids characterizing the samples 4-ΒΒ and 6P-BCP, while in 1-BB, they were present with lower intensity (in comparison to the rest of the polyphenols). Samples 4-ΒΒ and 6P-BCP possess a similar chemical polyphenolic profile in addition to 1-BB, whereas additional predominant signals indicated the presence of pinocembrin, pinobanksin, and chrysin-related components [38,39]. Other phenolics derivatives, such as simple phenolic isomers, were also detected as minor constituents. The glycosidic content analysis revealed different types of free sugars and/or sugar moieties of glucosides in all samples. Besides monosaccharides such as α- and β- glucose, other carbohydrates, such as fructose and sucrose, maltose, and inositol derivatives, were detected. Of note, under these conditions, 1-BB-Bu demonstrates a lower glycosidic content based on the lower signal intensity of the anomeric protons, while in 4-ΒΒ-Bu and 6P-BCP-Bu, the sugars are present in equal proportions. Concerning hydrocarbon and lipid accumulation, their presence was confirmed in all samples. Moreover, the GC-MS analyses of 1-BB-Et, 4-BB-Et and 6P-BCP-Et unveiled the presence of n-alkanes in all samples, including tricosane and pentacosane. A detailed discussion of the NMR and GC-MS data is provided in Appendix A and Supplementary Material along with related 1D-/2D-NMR and GC-MS chromatograms (Figure A1 and Figures S13–S15).

## 4. Conclusions

Overall, BCP, BB and artificially fermented BCP exerted significant antiviral activity against influenza A virus (IAV) H1N1. Artificially fermented BCP samples AF5 and AF17 demonstrated higher SI values, indicating a putative contribution of the BB microbiome to the recorded antiviral activity. The proteinaceous fractions of BCP, BB and artificially fermented BB demonstrated the highest SI values, indicating the proteinaceous nature of unknown antiviral agent(s), which may synergistically act with antiviral-plant-derived metabolites identified in the chemical profile of BCP and BB samples. NMR and LC-MS analyses revealed the presence of known antiviral metabolites such as quercetin, which may contribute toward antiviral activity. Furthermore, the NMR and LC-MC fingerprints provide a reliable methodology of revealing metabolomic patterns that might be correlated to observed biological effects. To the best of our knowledge, few studies based on NMR and LC-MS have been conducted regarding the evaluation of bee-pollen-derived products. Of note, by enriching the literature with chemical analysis reports of diverse sets of samples, the data interpretation will become increasingly applicable, thus providing faster and higher quality results. Nevertheless, further research is warranted regarding the antiviral potential of BCP and BB, their putative mode of action, as well as their potential synergy effects, in view of novel treatments against IAV H1N1.

## Figures and Tables

**Table 1:** Description of samples tested in this study.

Sample	Type	Description
AF1	Bee collected pollen	Multifloral Thessaly, Greece, Spring 2022
AF4	Fermented AF1 BCP	
AF5	Fermented AF1 BCP	
AF17	Fermented AF1 BCP	
AF1-P	Bee collected pollen	Proteinaceous fraction of AF1
AF4-P	Fermented BCP fraction	Proteinaceous fraction of AF4
AF5-P	Fermented BCP fraction	Proteinaceous fraction of AF5
AF17-P	Fermented BCP fraction	Proteinaceous fraction of AF17
1-BB	Beebread	Multifloral Thessaly, Greece, Spring 2019
4-BB	Beebread	Multifloral Thessaly, Greece, Spring 2019
6P-BCP	Bee-collected pollen	Multifloral Thessaly, Greece, Spring 2019
1-BB-P	Beebread fraction	Proteinaceous fraction of 1-BB
4-BB-P	Beebread fraction	Proteinaceous fraction of 4-BB
6P-BCP-P	Bee-collected pollen fraction	Proteinaceous fraction of 6-BCP
1-BB-Bu	Beebread fraction	*n*-butanol fraction of 1-BB
4-BB-Bu	Beebread fraction	*n*-butanol fraction of 4-BB
6P-BCP-Bu	Bee-collected pollen fraction	*n*-butanol fraction of 6-BCP
4-BB-H	Beebread fraction	Aqueous fraction of 4-BB
6P-BCP-H	Bee-collected pollen fraction	Aqueous fraction of 6-BCP

**Table 2:** Antiviral activity of tested samples. CC_50_ and IC_50_ values are shown as mg/mL.

Scheme 50	CC_50_	IC_50_	SI
AF1	11.57	3.91	2.95
AF4	10.63	10.04	1.06
AF5	11.11	2.27	4.89
AF17	12.00	2.47	4.85
AF1-P	>64	3.14	20.38
AF4-P	51.18	2.71	18.88
AF5-P	47.78	3.37	14.17
AF17-P	>64	0.46	139.13
1-BB	4.88	0.16	31.28
4-BB	0.83	0.18	4.60
6P-BCP	0.47	0.42	1.12
1-BB-P	32.00	0.097	329.89
4-BB-P	7.45	0.022	338.64
6P-BCP-P	3.92	0.052	75.38
1-BB-Bu	>64	2.91	21.99
4-BB-Bu	>64	-	-
6P-BCP-Bu	>64	-	-
4-BB-H	7.70	-	-
6P-BCP-H	6.97	-	-

AF: artificially fermented sample; P: proteinaceous fraction; Bu: *n*-butanol fraction; H: aqueous fraction; SI: Selectivity Index.

**Table 3:** LC-MS analysis of *n*-butanol fractions (1-BB-Bu, 4-BB-Bu and 6P-BCP-Bu samples).

Rt	Positive Ion Mode	Negative Ion Mode	Mass	Molecular Formula	Proposed Compounds	1-BB-Bu	4-BB-Bu	6P-BCP-Bu
	Found	Found						
0.324	203.0529 [M+Na]^+^	179.0564 [M−H]^−^	180.0634	C_6_H_12_O_6_	carbohydrates	•	•	•
0.337	365.1056 [M+Na]^+^	341.1091 [M−H]^−^	342.1165	C_12_H_22_O_11_	carbohydrates	•	•	•
0.490	349.1118 [M+Na]^+^	371.1186 [M+HCOO]^−^	326.1215	C_12_H_22_O_10_	carbohydrates	•	•	•
0.493	139.0390 [M+Na]^+^		138.0318	C_7_H_6_O_3_	simple phenolic	•	•	
0.680		134.0470 [M−H]^−^	135.0542	C_4_H_9_NO_4_	amino acid derivative	•	•	•
1.048	132.1018 [M+H]^+^			C_6_H_13_NO_2_	leucine isomer	•	•	•
1.730		243.0620 [M−H]^−^	244.0695	C_9_H_12_N_2_O_6_	uridine isomer	•		•
1.850	166.0864 [M+H]^+^		165.079	C_9_H_11_NO_2_	amino acid	•	•	•
1.998	149.0241 [M+H]^+^		148.1583	C_9_H_8_O_2_	cinnamic acid isomer	•		
2.046	181.0498 [M+H]^+^	179.0350 [M−H]^−^	180.0421	C_9_H_8_O_4_	caffeic acid isomer	•		•
2.426	195.0649 [M+H]^+^		194.0579,	C_10_H_10_O_4_	ferulic acid isomer	•	•	
3.210	153.0546 [M+H]^+^	151.0400 [M−H]^−^	152.0468	C_8_H_8_O_3_	methoxybenzoic acid	•	•	
3.772	181.0496 [M+H]^+^	179.0349 [M−H]^−^	180.0421	C_9_H_8_O_4_	caffeic acid isomer	•		•
4.936	627.1563 [M+H] +	625.141 [M−H]^−^	626.1482	C_27_H_30_O_17_	flavonoid di-glycoside	•	•	•
4.981	611.1611 [M+H]^+^	595.1303 [M−H]^−^	609.1462	C_27_H_30_O_16_	flavonoid di-glycoside	•	•	•
5.000	641.1713 [M+H]^+^	639.1562 [M−H]^−^	640.1633	C_28_H_32_O_17_	flavonoid di-glycoside	•		•
5.078	641.1721 [M+H]^+^	639.1570 [M−H]^−^	640.1646	C_28_H_32_O_17_	flavonoid di-glycoside	•		•
5.182	597.1451 [M+H]^+^	595.1300 [M−H]^−^	596.1377	C_26_H_28_O_16_	flavonoid di-glycoside	•		•
5.191	641.1722 [M+H]^+^	639.1568 [M−H]^−^	640.1648	C_28_H_32_O_17_	flavonoid di-glycoside		•	
5.200	671.1820 [M+H]^+^	669.1665 [M−H]^−^	670.1739	C_29_H_34_O_18_	flavonoid di-glycoside			•
5.260	597.1460 [M+H]^+^	595.1303 [M−H]^−^	596.1379	C_26_H_28_O_16_	flavonoid di-glycoside		•	•
5.266	773.2135 [M+H]^+^	771.1986 [M−H]^−^	772.2062	C_33_H_40_O_21_	flavonoid tri-glycoside	•		•
5.320	611.1612 [M+H]^+^	609.1465 [M−H]^−^	610.1539	C_27_H_30_O_16_	flavonoid di-glycoside		•	•
5.440	787.2298 [M+H]^+^	785.2148 [M−H]^−^	786.2224	C_34_H_42_O_21_	flavonoid tri-glycoside	•		•
5.460	611.1618 [M+H]^+^	609.1452 [M−H]^−^	610.1525	C_27_H_30_O_16_	flavonoid di-glycoside	•		
5.531	625.1763 [M+H]^+^	623.1616 [M−H]-	624.1691	C_28_H_32_O_16_	flavonoid tri-glycoside		•	
5.570	611.1621 [M+H]^+^	609.1455 [M−H]^−^	610.1529	C_27_H_30_O_16_	flavonoid di-glycoside	•	•	
5.603	563.1759 [M+H]^+^	561.1614 [M−H]^−^	562.1686	C_27_H_30_O_13_	flavonoid di-glycoside	•		
5.647	757.2190 [M+H]^+^	755.2041 [M−H]^−^	756.2116	C_33_H_40_O_20_	flavonoid tri-glycoside			•
5.741	595.1659 [M+H]^+^	593.1511 [M−H]^−^	594.1585	C_27_H_30_O_15_	flavonoid di-glycoside	•	•	•
5.790	581.1506 [M+H]^+^	579.1351 [M−H]^−^	580.1433	C_26_H_28_O_15_	flavonoid di-glycoside		•	•
5.840	625.1770 [M+H]^+^	623.1616 [M−H]^−^	624.1696	C_28_H_32_O_16_	flavonoid di-glycoside	•	•	•
5.881	611.1617 [M+H]^+^	609.1454 [M−H]^−^	610.1527	C_27_H_30_O_16_	flavonoid di-glycoside		•	•
6.258	625.1768 [M+H]^+^	623.1610 [M−H]^−^	624.1682	C_28_H_32_O_16_	flavonoid di-glycoside	•		
6.355	449.1078 [M+H]^+^	447.0930 [M−H]^−^	448.1005	C_21_H_20_O_11_	flavonoid glycoside	•		•
6.382	417.1008 [M+H]^+^	415.1033 [M−H]^−^	416.3789	C_21_H_20_O_9_	flavonoid glycoside	•		
6.751	465.1028 [M+H]^+^	463.0946 [M−H]^−^	464.0955	C_21_H_20_O_12_	flavonoid glycoside	•		•
7.033	433.1026 [M+H]^+^	431.0975 [M−H]^−^	432.1048	C_21_H_20_O_10_	flavonoid glycoside	•		
7.403	653.1714 [M+H]^+^	651.1561 [M−H]^−^	652.1642	C_29_H_32_O_17_	flavonoid tri-glycoside			•
7.812	303.0500 [M+H]^+^	301.0352 [M−H]^−^	302.0427	C_15_H_10_O_7_	quercetin	•		
7.841		255.0662 [M−H]^−^	256.2568	C_15_H_12_O_4_	isoflavone derivative	•		
8.936	317.0658 [M+H]^+^	315.0511 [M−H]^−^	316.0583	C_16_H_12_O_7_	methoxylated flavonoid		•	
8.995	287.0553 [M+H]^+^	285.0405 [M−H]^−^	286.0478	C_15_H_10_O_6_	kaempferol	•	•	•
8.999	255.0652 [M+H]^+^	253.0506 [M−H]^−^	254.2423	C_15_H_10_O_4_	isoflavone derivative	•		
9.230	317.0658 [M+H]^+^	315.0508 [M−H]^−^	316.0558	C_16_H_12_O_7_	methoxylated flavonoid		•	•

Shaded squares indicate the presence of chemical entities.

## Data Availability

Data is contained within the article or Supplementary Material.

## Supplementary Materials

The following supporting information can be downloaded at: https://www.mdpi.com/article/10.3390/foods12101978/s1, Figure S1: ^1^H NMR (400 MHz, CDCl_3_) spectrum of 1-BB-Et; Figure S2: ^1^H NMR (400 MHz, MeOD) spectrum of 1-BB-Bu; Figure S3: ^1^H-^1^H COSY (400 MHz, MeOD) spectrum of 1-BB-Bu; Figure S4: HSQC NMR (400 MHz, MeOD) spectrum 1-BB-Bu; Figure S5: HMBC NMR (400 MHz, MeOD) spectrum 1-BB-Bu; Figure S6: ^1^H NMR (CDCl_3_) spectrum 4-BB-Et; Figure S7: ^1^H NMR (MeOD) spectrum 4-BB-Bu; Figure S8: ^1^H NMR (600 MHz, CDCl_3_) of 6P-BCP-Et; Figure S9: ^1^H NMR (600 MHz, MeOD) of 6P-BCP-Bu; Figure S10: LC-ESI-MS of 1-BB-Bu extract in positive (A) and negative (B) ion modes; Figure S11: LC-ESI-MS of 4-BB-Bu extract in positive (A) and negative (B) ion modes; Figure S12: LC-ESI-MS of 6P-BCP-Bu extract in positive (A) and negative (B) ion modes; Figure S13: GC-MS chromatogram of 1-BB-Et; Figure S14: GC-MS chromatogram of 4-BB-Et; Figure S15: GC-MS chromatogram of 6P-BCP-Et.

## Appendix A. NMR Characterization of Selected Bee-Derived n-Butanol Residues

An overlay of the ^1^H-NMR spectra acquired for the under-investigation bee breads (1-ΒΒ-Bu, 4-ΒΒ-Bu) and bee-collected pollen (6P-BCP-Bu) samples is depicted in Figure A1. The regional differentiation of NMR resonance signals serves to illustrate the different chemical groups, where phenolic derivatives (blue background), carbohydrates/sugars, (orange background), and hydrocarbons and fatty acids signals (green background) have been highlighted in different colors.

In detail, visual inspection of the 1D and 2D NMR spectra revealed the presence of different classes of metabolites in accordance with the literature [8]. In the ^1^H NMR spectrum (in CD_3_OD), the different functional groups are indicated by the presence of characteristic resonances [22], whereas polyphenols in the form of aromatic protons were shifted at δ_H_ 8.3–5.5 ppm, followed by an overcrowded region in higher fields, at δ_H_ 5.5–2.8 ppm, with mainly proton signals assigned for different types of free sugars and sugar moieties of glucoside derivatives (such as phenolic glucosides). Lastly, in higher fields at δ_H_ 2.5–0.6 ppm, signals ascribable to aliphatic hydrocarbons and lipids were observed.

Starting with phenolic derivatives in all samples, flavonoids were detected. The ^1^H-NMR spectra exhibit signals belonging to olefinic protons of the characteristic positions H-8 and H-6 of the A-ring (of flavonoids) at δ_H_ 6.42–6.33 and 6.28–6.17, respectively [40]. Such signals especially characterized the samples 4-ΒΒ and 6P-BCP, while in 1-BB, they were also detected with lower intensity (in comparison with the rest of the polyphenols). Other phenolics derivatives, such as simple phenolic isomers, were also detected as minor components in this region and, on the bases of the signal pattern (doublets with J = 16.0 Hz for *trans* bond and J = 12.0 Hz for *cis* bond; ABX system with d J = 2.0 Hz, d J = 8.0 Hz and dd J = 8.0, 2.0 Hz), were attributed to hydroxycinnamic derivatives. Samples 4-ΒΒ and 6P-BCP possess a similar chemical polyphenolic profile in addition to 1-BB, whereas additional predominant phenolics cover the region at δ_H_ 7.40–6.20. Such signals are characteristic of flavonoids bearing a B-ring without any substituent, such as pinocembrin, pinobanksin, chrysin, etc. [38,39].

In higher fields and especially the region 5.5–2.8 ppm, analysis of the glycosidic content was allowed. Signals for different types of free sugars and/or sugar moieties of glucosides appear as depicted in Figure A1 in all samples. At δ_H_ 5.10 (J = 3.8 Hz) and 4.47 (J = 8.0 Hz), two doublets respond to the anomeric protons of free α- and β- glucose, respectively [41]. Besides the above-mentioned monosaccharides, other carbohydrates, such as fructose and sucrose, maltose, and inositol derivatives, have been documented through the 2D NMR experiments due to the overlapped signals in ^1^H-NMR [42]. Moreover, under the given conditions, 1-BB possesses a lower glycosidic content based on the lower signal intensity of the anomeric protons, while in 4-ΒΒ and 6P-BCP, the sugars were present in equal proportions.

Concerning hydrocarbon and lipid accumulation, their presence was confirmed in all samples (see ^1^H-NMR in CDCl_3_). The multiplets at δ_H_ ca. 5.35–5.30 were attributed to the olefinic protons (-CH = CH-) of the unsaturated aliphatic chain, while the triplets at δ_H_ ca. 0.88 (J = 6.9) and/or 0.95 (J = 6.9) were attributed to the terminal CH_3_ group of the chain, depending on the degree of unsaturation. The nearby -CH_2_ of the esters (-COOR) were distributed at δ_H_ ca. 2.33 (t, J = 6.8), and the intermediate -CH_2_- of the double bonds were resonated at δ_H_ ca. 2.76 (m). The rest (-CH_2_)_n_ of the chains were assigned to the intense signal in higher fields (δ_H_ ca. 1.24) [22]. Moreover, the GC-MS analyses unveiled the presence of n-alkanes in all samples, i.e., tricosane (C_23_H_48_) and pentacosane (C_25_H_52_).

## References

[1] Mărgăoan R., Stranț M., Varadi A., Topal E., Yücel B., Cornea-Cipcigan M., Campos M.G., Vodnar D.C. (2019). Bee Collected Pollen and Bee Bread: Bioactive Constituents and Health Benefits. Antioxidants.

[2] Kostić A.Ž., Milinčić D.D., Barać M.B., Ali Shariati M., Tešić Ž.L., Pešić M.B. (2020). The Application of Pollen as a Functional Food and Feed Ingredient—The Present and Perspectives. Biomolecules.

[3] El Ghouizi A., Bakour M., Laaroussi H., Ousaaid D., El Menyiy N., Hano C., Lyoussi B. (2023). Bee Pollen as Functional Food: Insights into Its Composition and Therapeutic Properties. Antioxidants.

[4] Laaroussi H., Ferreira-Santos P., Genisheva Z., Bakour M., Ousaaid D., El Ghouizi A., Teixeira J.A., Lyoussi B. (2023). Unveiling the techno-functional and bioactive properties of bee pollen as an added-value food ingredient. Food Chem..

[5] Didaras N.A., Karatasou K., Dimitriou T.G., Amoutzias G.D., Mossialos D. (2020). Antimicrobial Activity of Bee-Collected Pollen and Beebread: State of the Art and Future Perspectives. Antibiotics.

[6] Vásquez A., Olofsson T.C. (2009). The lactic acid bacteria involved in the production of bee pollen and bee bread. J. Apic. Res..

[7] Didaras N.A., Kafantaris I., Dimitriou T.G., Mitsagga C., Karatasou K., Giavasis I., Stagos D., Amoutzias G.D., Hatjina F., Mossialos D. (2021). Biological Properties of Bee Bread Collected from Apiaries Located across Greece. Antibiotics.

[8] Bakour M., Laaroussi H., Ousaaid D., El Ghouizi A., Es-Safi I., Mechchate H., Lyoussi B. (2022). Bee Bread as a Promising Source of Bioactive Molecules and Functional Properties: An Up-To-Date Review. Antibiotics.

[9] Pełka K., Otłowska O., Worobo R.W., Szweda P. (2021). Bee Bread Exhibits Higher Antimicrobial Potential Compared to Bee Pollen. Antibiotics.

[10] Kontogiannis T., Dimitriou T.G., Didaras N.A., Mossialos D. (2022). Antiviral Activity of Bee Products. Curr. Pharm. Des..

[11] Asma S.T., Bobiş O., Bonta V., Acaroz U., Shah S.R.A., Istanbullugil F.R., Arslan-Acaroz D. (2022). General Nutritional Profile of Bee Products and Their Potential Antiviral Properties against Mammalian Viruses. Nutrients.

[12] Didaras N.A., Dimitriou T.G., Daskou M., Karatasou K., Mossialos D. (2022). In Vitro Assessment of the Antiviral Activity of Greek Bee Bread And Bee Collected Pollen Against Enterovirus D68. J. Microbiol. Biotechnol. Food Sci..

[13] Lee I.-K., Hwang B., Kim D.-W., Kim J.-Y., Woo E.-E., Lee Y.-J., Choi H., Yun B.-S. (2016). Characterization of Neuraminidase Inhibitors in Korean Papaver rhoeas Bee Pollen Contributing to Anti-Influenza Activities In Vitro. Planta Med..

[14] Chauhan R.P., Gordon M.L. (2022). An overview of influenza A virus genes, protein functions, and replication cycle highlighting important updates. Virus Genes.

[15] Wang J., Sun Y., Liu S. (2022). Emerging antiviral therapies and drugs for the treatment of influenza. Expert Opin. Emerg. Drugs.

[16] Govorkova E.A., Takashita E., Daniels R.S., Fujisaki S., Presser L.D., Patel M.C., Huang W., Lackenby A., Nguyen H.T., Pereyaslov D. (2022). Global update on the susceptibilities of human influenza viruses to neuraminidase inhibitors and the cap-dependent endonuclease inhibitor baloxavir, 2018–2020. Antivir. Res..

[17] Pfannebecker J., Schiffer-Hetz C., Fröhlich J., Becker B. (2016). Culture medium optimization for osmotolerant yeasts by use of a parallel fermenter system and rapid microbiological testing. J. Microbiol. Methods.

[18] Tsadila C., Nikolaidis M., Dimitriou T.G., Kafantaris I., Amoutzias G.D., Pournaras S., Mossialos D. (2021). Antibacterial Activity and Characterization of Bacteria Isolated from Diverse Types of Greek Honey against Nosocomial and Foodborne Pathogens. Appl. Sci..

[19] Kumar S., Stecher G., Li M., Knyaz C., Tamura K. (2018). MEGA X: Molecular Evolutionary Genetics Analysis across Computing Platforms. Mol. Biol. Evol..

[20] Wingfield P. (1998). Protein Precipitation Using Ammonium Sulfate. Current Protocols in Protein Science.

[21] Acar Şahin A., Aslım B., Tan S., Alan Ş., Pınar N.M. (2018). Differences in structure, allergenic protein content and pectate lyase enzyme activity of some Cupressaceae pollen. Turk. J. Biochem..

[22] Tsami K., Barda C., Ladopoulos G., Didaras N.A., Grafakou M.-E., Heilmann J., Mossialos D., Rallis M.C., Skaltsa H. (2022). Chemical Profile and In Vitro Evaluation of the Antibacterial Activity of *Dioscorea communis* Berry Juice. Sci.

[23] van Meerloo J., Kaspers G.J.L., Cloos J., Cree I. (2011). Cell Sensitivity Assays: The MTT Assay. Cancer Cell Culture. Methods in Molecular Biology (Methods and Protocols).

[24] Casas I., Powell L., Klapper P.E., Cleator G.M. (1995). New method for the extraction of viral RNA and DNA from cerebrospinal fluid for use in the polymerase chain reaction assay. J. Virol. Methods.

[25] Eisfeld A.J., Neumann G., Kawaoka Y. (2014). Influenza A virus isolation, culture and identification. Nat. Protoc..

[26] Pritchett J.C., Naesens L., Montoya J., Flamand L., Lautenschlager I., Krueger G.R.F., Ablashi D.V. (2014). Treating HHV-6 Infections: The Laboratory Efficacy and Clinical Use of Anti-HHV-6 Agents. Human Herpesviruses HHV-6A, HHV-6B, and HHV-7.

[27] Cavalli R., Donalisio M., Bisazza A., Civra A., Ranucci E., Ferruti P., Lembo D., Düzgüneş N. (2012). Enhanced Antiviral Activity of Acyclovir Loaded into Nanoparticles. Methods in Enzymology.

[28] Di Cagno R., Filannino P., Cantatore V., Gobbetti M. (2019). Novel solid-state fermentation of bee-collected pollen emulating the natural fermentation process of bee bread. Food Microbiol..

[29] Kaškonienė V., Adaškevičiūtė V., Kaškonas P., Mickienė R., Maruška A. (2020). Antimicrobial and antioxidant activities of natural and fermented bee pollen. Food Biosci..

[30] Adaškevičiūtė V., Kaškonienė V., Barčauskaitė K., Kaškonas P., Maruška A. (2022). The Impact of Fermentation on Bee Pollen Polyphenolic Compounds Composition. Antioxidants.

[31] Gilliam M. (1979). Microbiology of pollen and bee bread: The yeasts. Apidologie.

[32] Saksinchai S., Suzuki M., Lumyong S., Ohkuma M., Chantawannakul P. (2012). Two new species of the genus *Candida* in the *Zygoascus* clade, *Candida lundiana* sp. nov. and *Candida suthepensis* sp. nov., isolated from raw honey in Thailand. Antonie Van Leeuwenhoek.

[33] Poyraz F., Yalmanci D., İspirli H., Dertli E. (2023). Characterization of Bee Bread Produced with Defined Starter Cultures Mimicking the Natural Fermentation Process. Fermentation.

[34] Gilliam M. (1979). Microbiology of pollen and bee bread: The genus *Bacillus*. Apidologie.

[35] Pełka K., Worobo R.W., Walkusz J., Szweda P. (2021). Bee Pollen and Bee Bread as a Source of Bacteria Producing Antimicrobials. Antibiotics.

[36] Watanabe K., Rahmasari R., Matsunaga A., Haruyama T., Kobayashi N. (2014). Anti-influenza Viral Effects of Honey In Vitro: Potent High Activity of Manuka Honey. Arch. Med. Res..

[37] Barda C., Anastasiou K., Tzara A., Grafakou M.-E., Kalpoutzakis E., Heilmann J., Rallis M., Kourounakis A.P., Skaltsa H. (2022). A Bio-Guided Screening for Antioxidant, Anti-Inflammatory and Hypolipidemic Potential Supported by Non-Targeted Metabolomic Analysis of *Crepis* spp.. Molecules.

[38] Peršurić Ž., Pavelić S.K. (2021). Bioactives from Bee Products and Accompanying Extracellular Vesicles as Novel Bioactive Components for Wound Healing. Molecules.

[39] Alotaibi M., Ali A., Bakhshwin D., Alatawi Y., Alotaibi S., Alhifany A., Alharthi B., Alharthi N., Alyazidi A., Alharthi Y. (2021). Effectiveness and Safety of Favipiravir Compared to Hydroxychloroquine for Management of Covid-19: A Retrospective Study. Int. J. Gen. Med..

[40] Mabry T.J., Markham K.R., Harborne J.B., Mabry T.J., Mabry H. (1975). Mass Spectrometry of Flavonoids BT. The Flavonoids.

[41] Papachristoforou A., Termentzi A., Halabalaki M., Koutouvela E. (2013). The application of highly centrifuged honey as an improved diet for experimentally caged honey bees. J. Apic. Res..

[42] Mazzei P., Piccolo A., Brescia M., Caprio E. (2020). Assessment of geographical origin and production period of royal jelly by NMR metabolomics. Chem. Biol. Technol. Agric..

