# Supplementary material for: Antiviral Activity of Beebread, Bee-Collected Pollen and Artificially Fermented Follen against Influenza A Virus
Source: Foods. 2023 May 13;12(10):1978. doi: 10.3390/foods12101978 (PMC10217140; doi:10.3390/foods12101978)
Supplement: Supplementary file 1 [file foods-12-01978-s001.zip › foods-2368483-supplementary.pdf]

S.1. NMR Spectra of 1-BB, 4-BB and 6P-BCP fractions

S.1.1. NMR Spectra of 1-BB

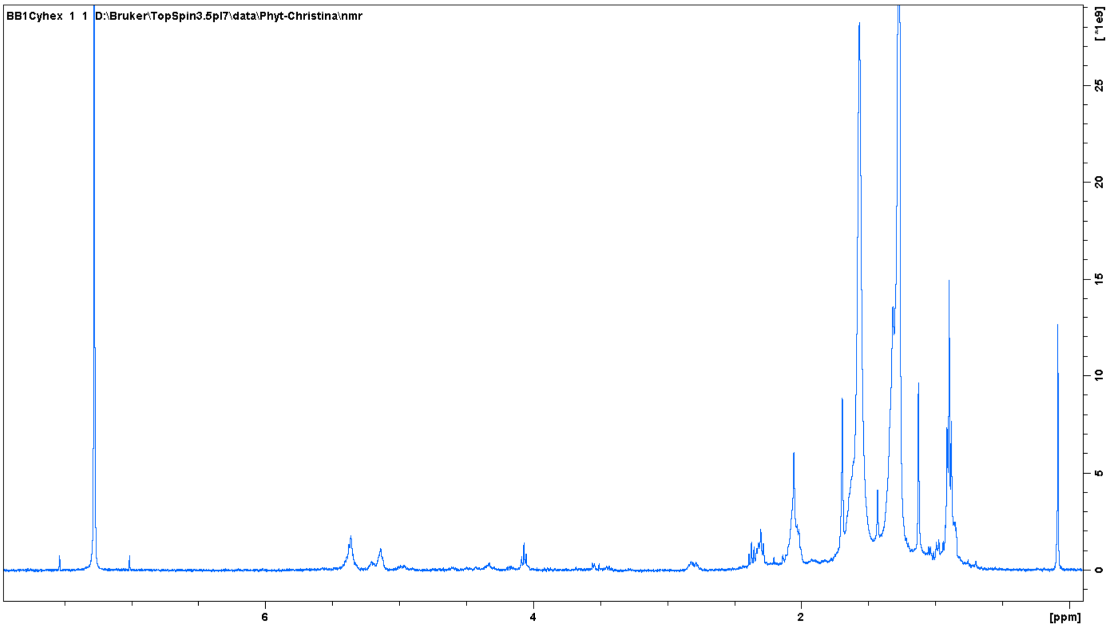

Figure S1. ^{1}H NMR (400 MHz, $CDCl_3$) spectrum of 1-BB-Et.

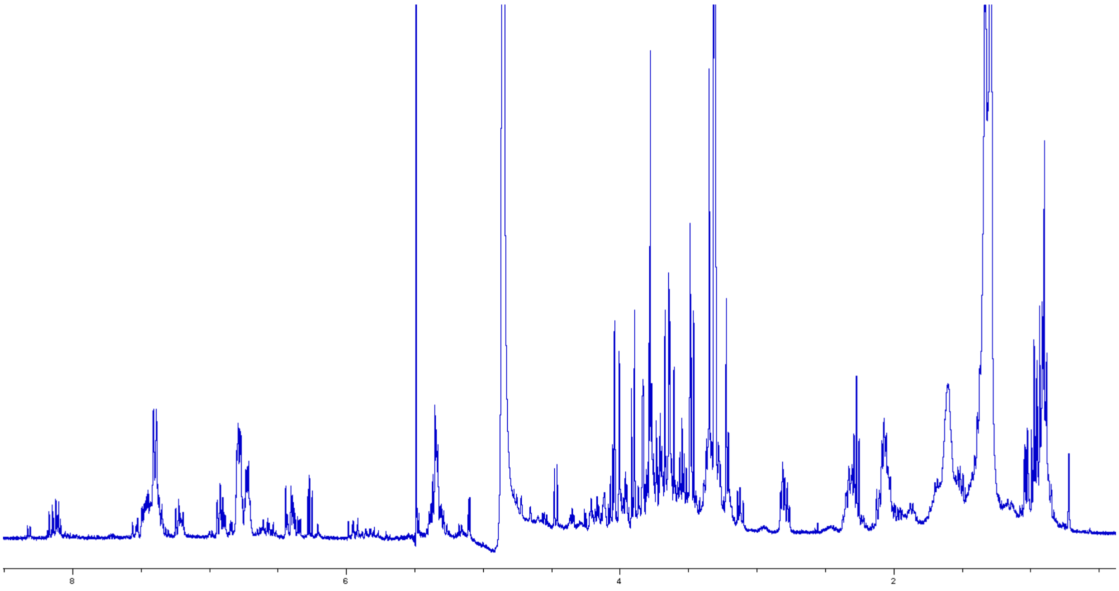

Figure S2. ^{1}H NMR (400 MHz, MeOD) spectrum of 1-BB-Bu.

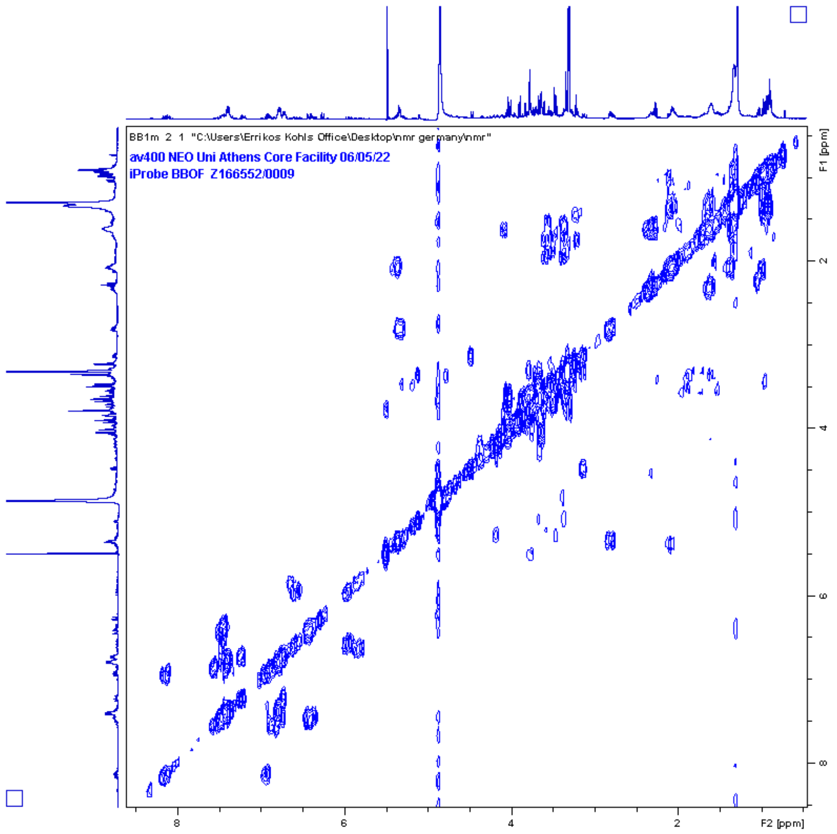

Figure S3. ^{1}H-^{1}H COSY (400 MHz, MeOD) spectrum of 1-BB-Bu.

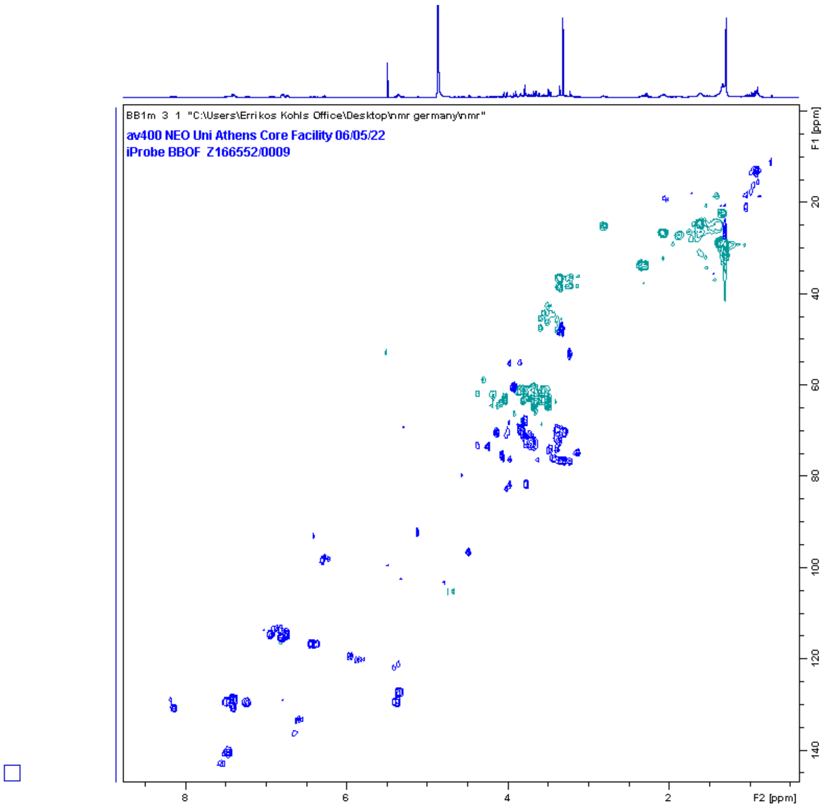

Figure S4. HSQC NMR (400 MHz, MeOD) spectrum 1-BB-Bu.

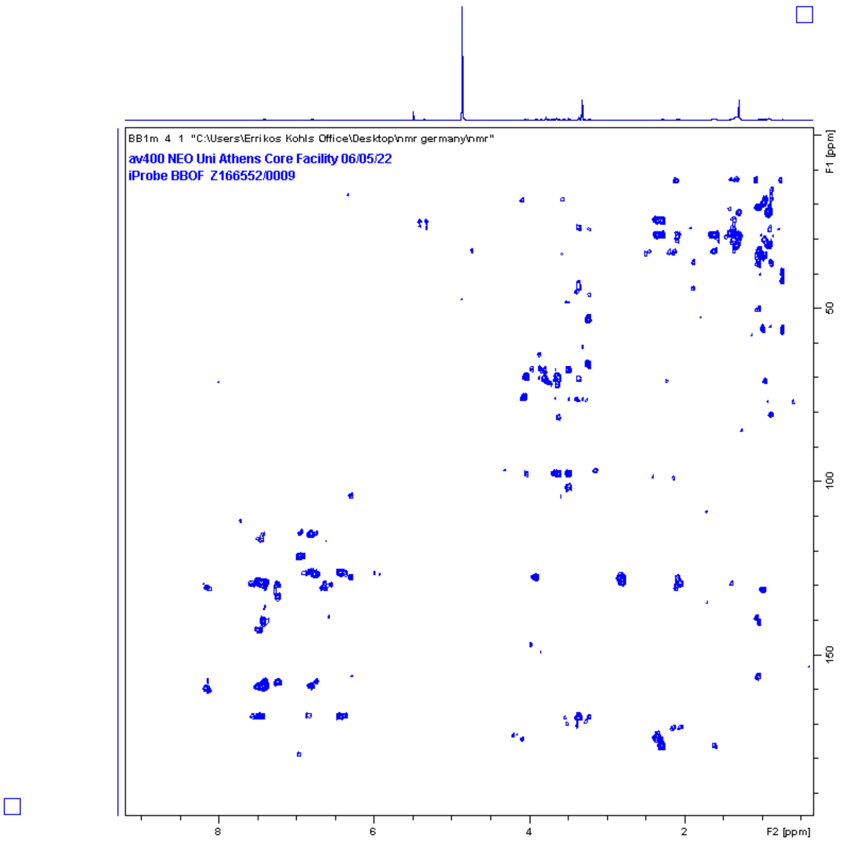

Figure S5. HMBC NMR (400 MHz, MeOD) spectrum 1-BB-Bu.

S.1.2. NMR spectra of 4-BB

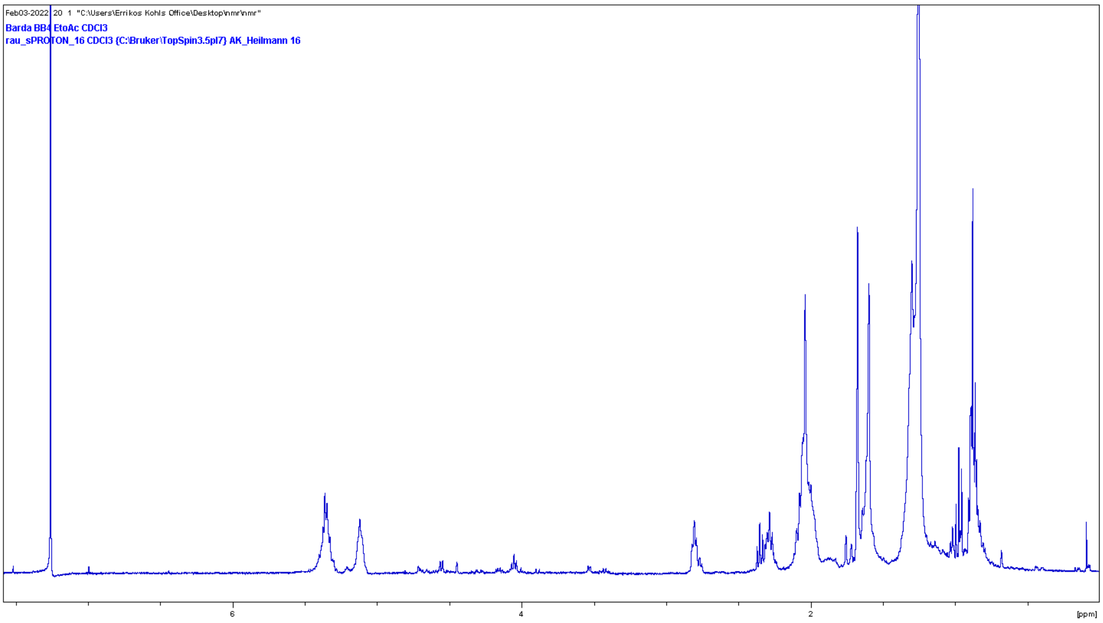

Figure S6. ^{1}H NMR ($CDCl_3$) spectrum 4-BB-Et.

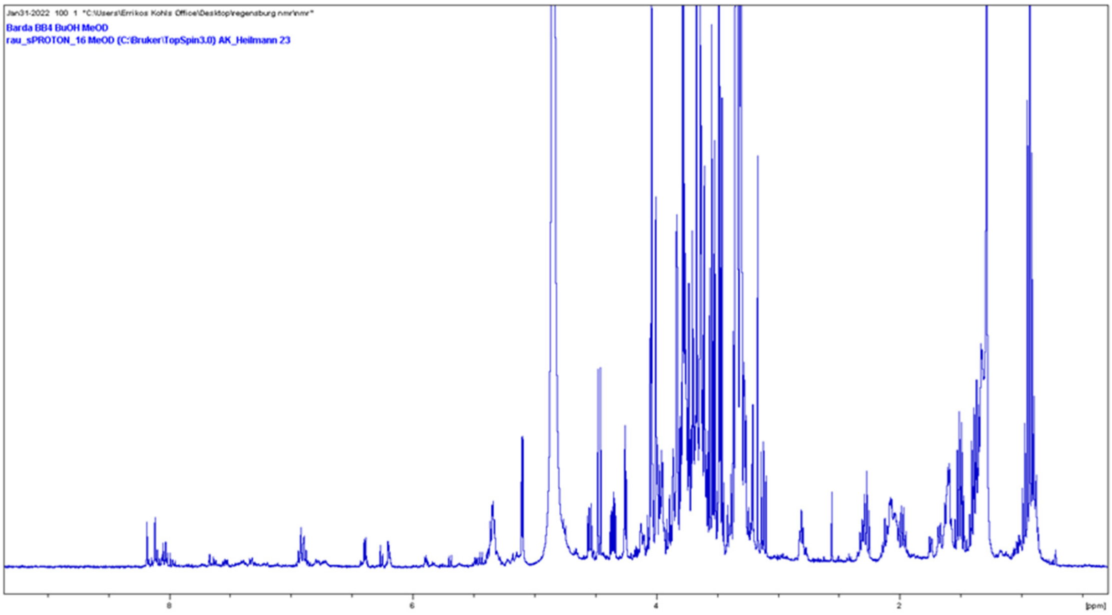

Figure S7. ^{1}H NMR (MeOD) spectrum 4-BB-Bu.

S.1.3. NMR spectra of 6P-BCP

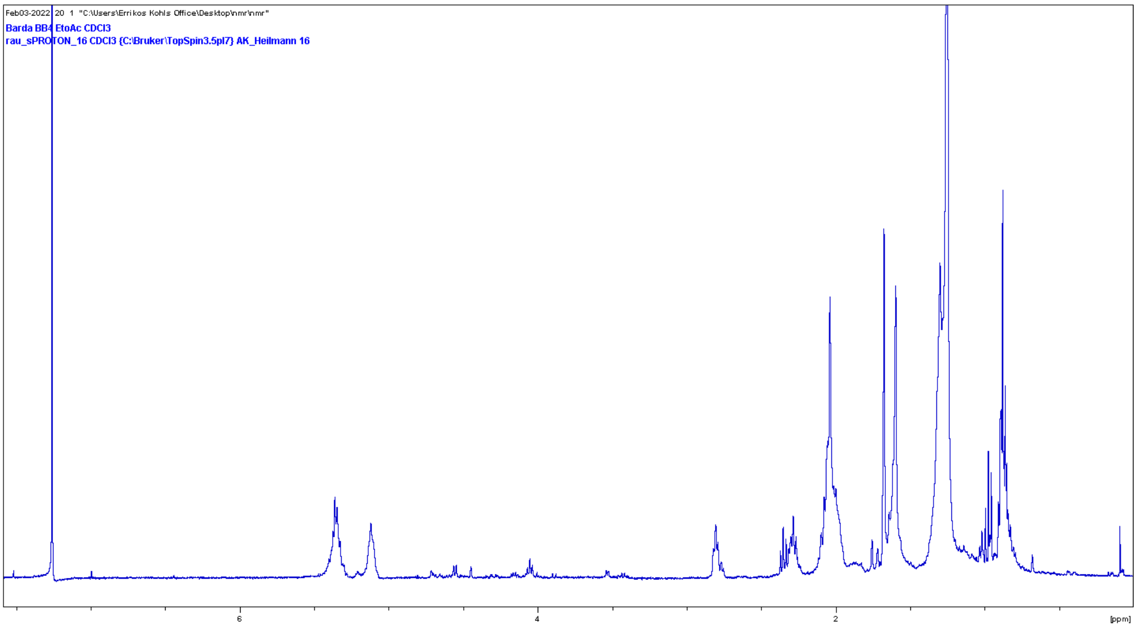

Figure S8. ^{1}H NMR (600 MHz, $CDCl_3$) of 6P-BCP-Et.

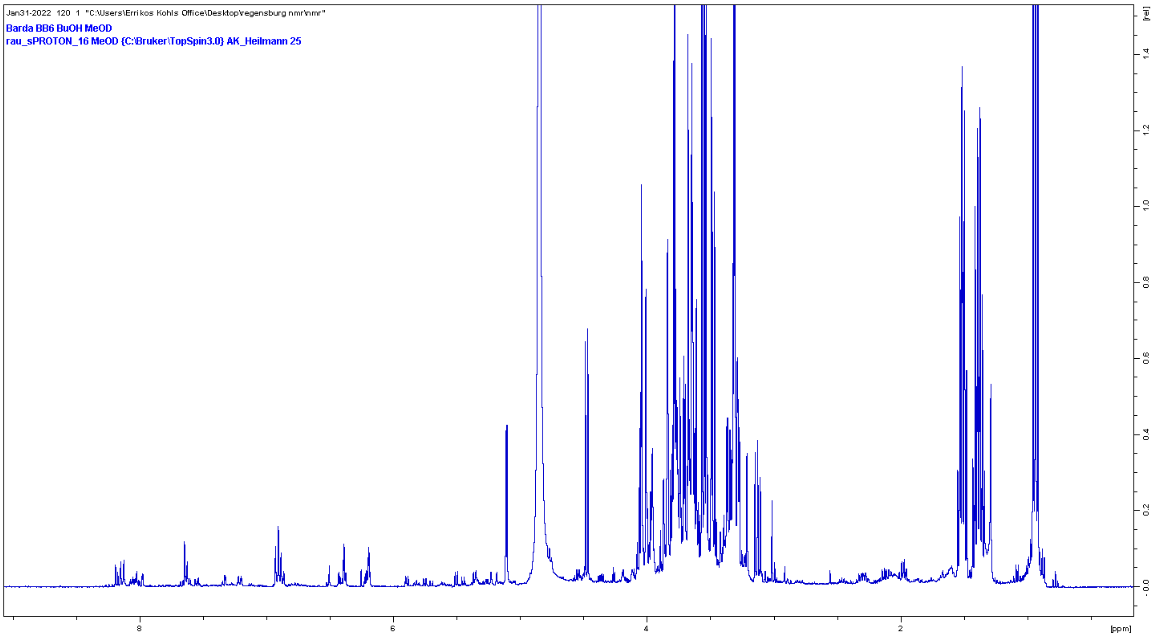

Figure S9. ^{1}H NMR (600 MHz, MeOD) of 6P-BCP-Bu.

S.2. LC-MS chromatograms of 1-BB-Bu, 4-BB-Bu and 6P-BCP-Bu

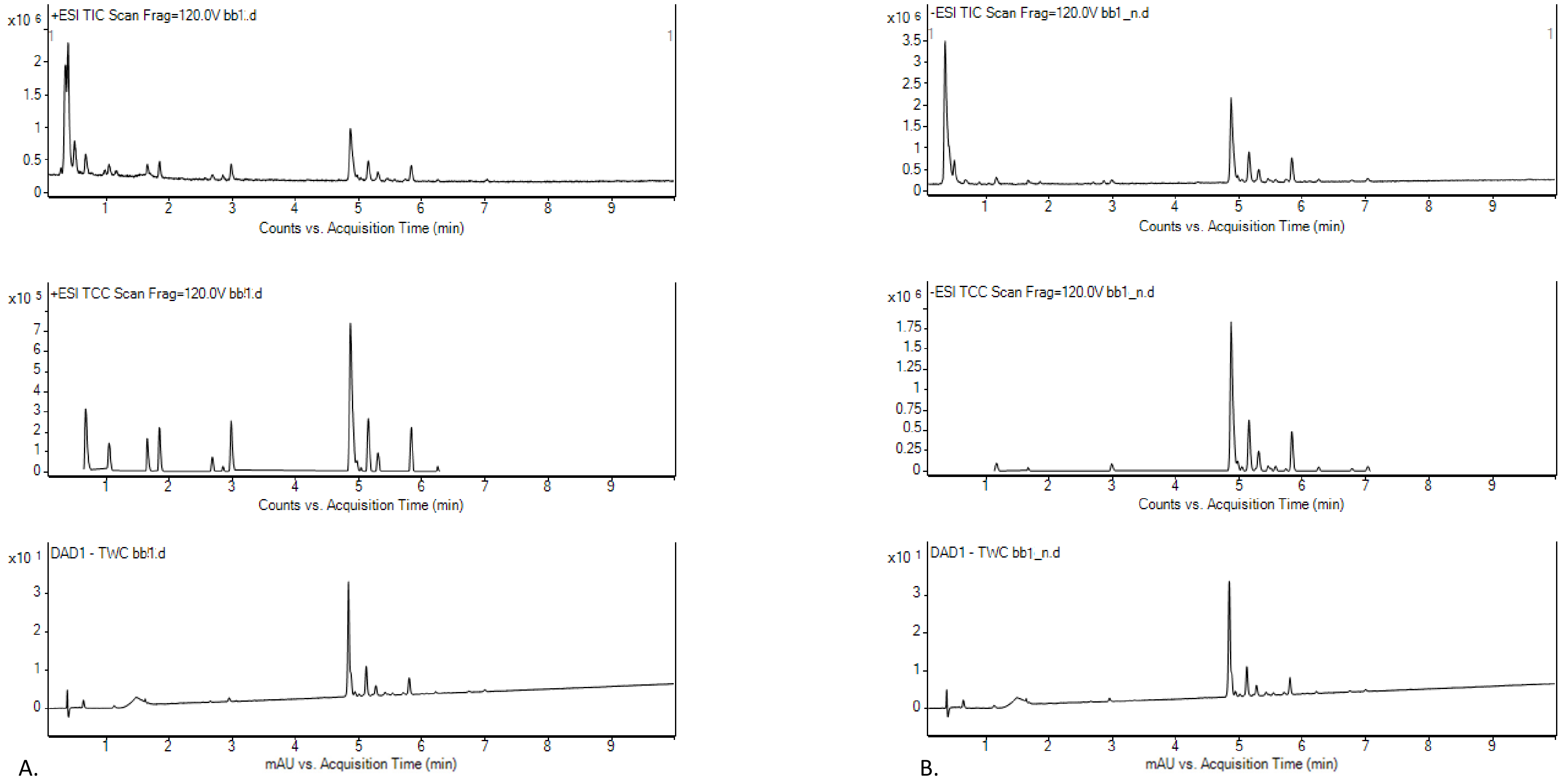

Figure S10. LC-ESI-MS of 1-BB-Bu extract in positive (A) and negative (B) ion modes.

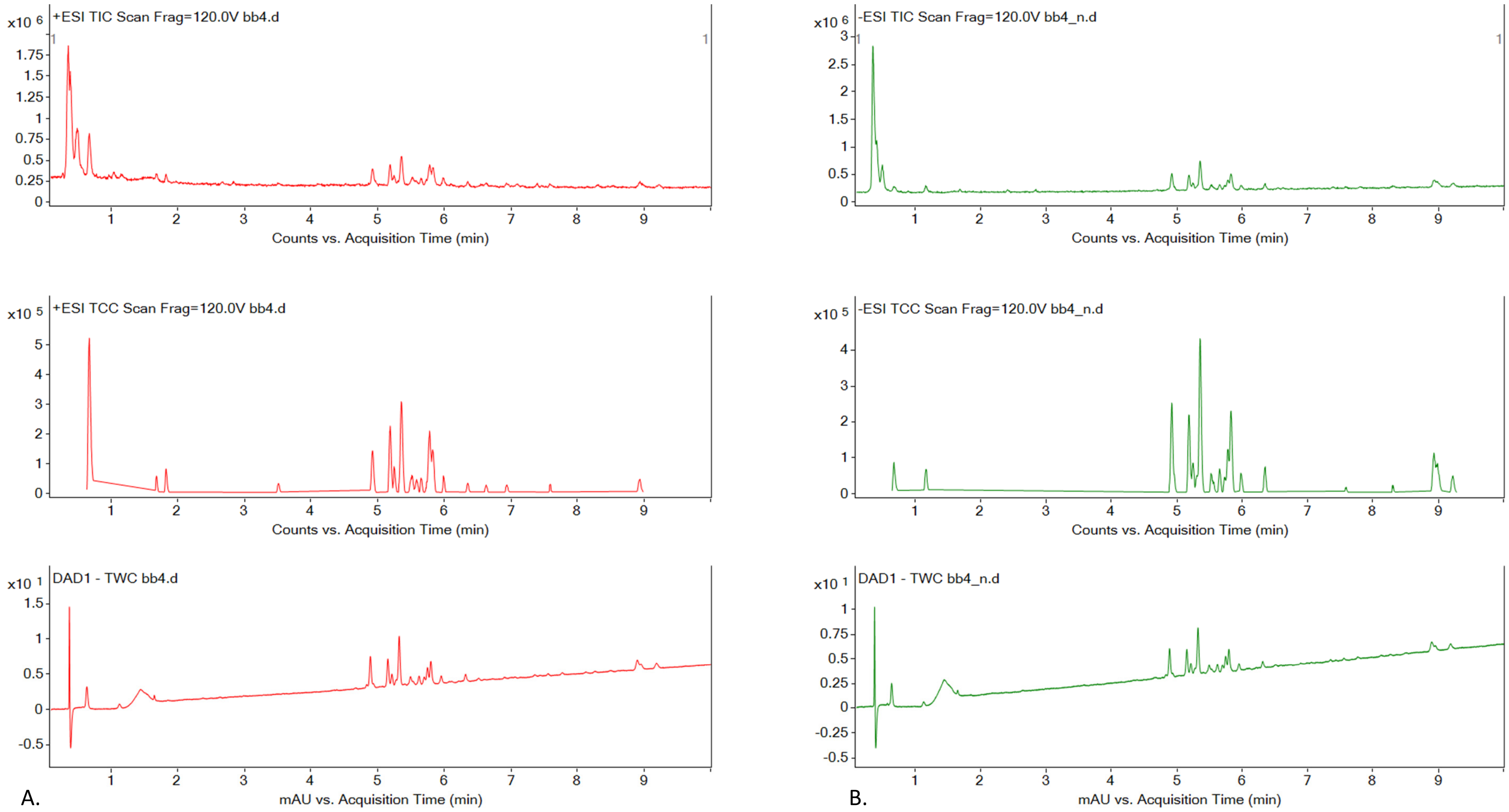

Figure S11. LC-ESI-MS of 4-BB-Bu extract in positive (A) and negative (B) ion modes.

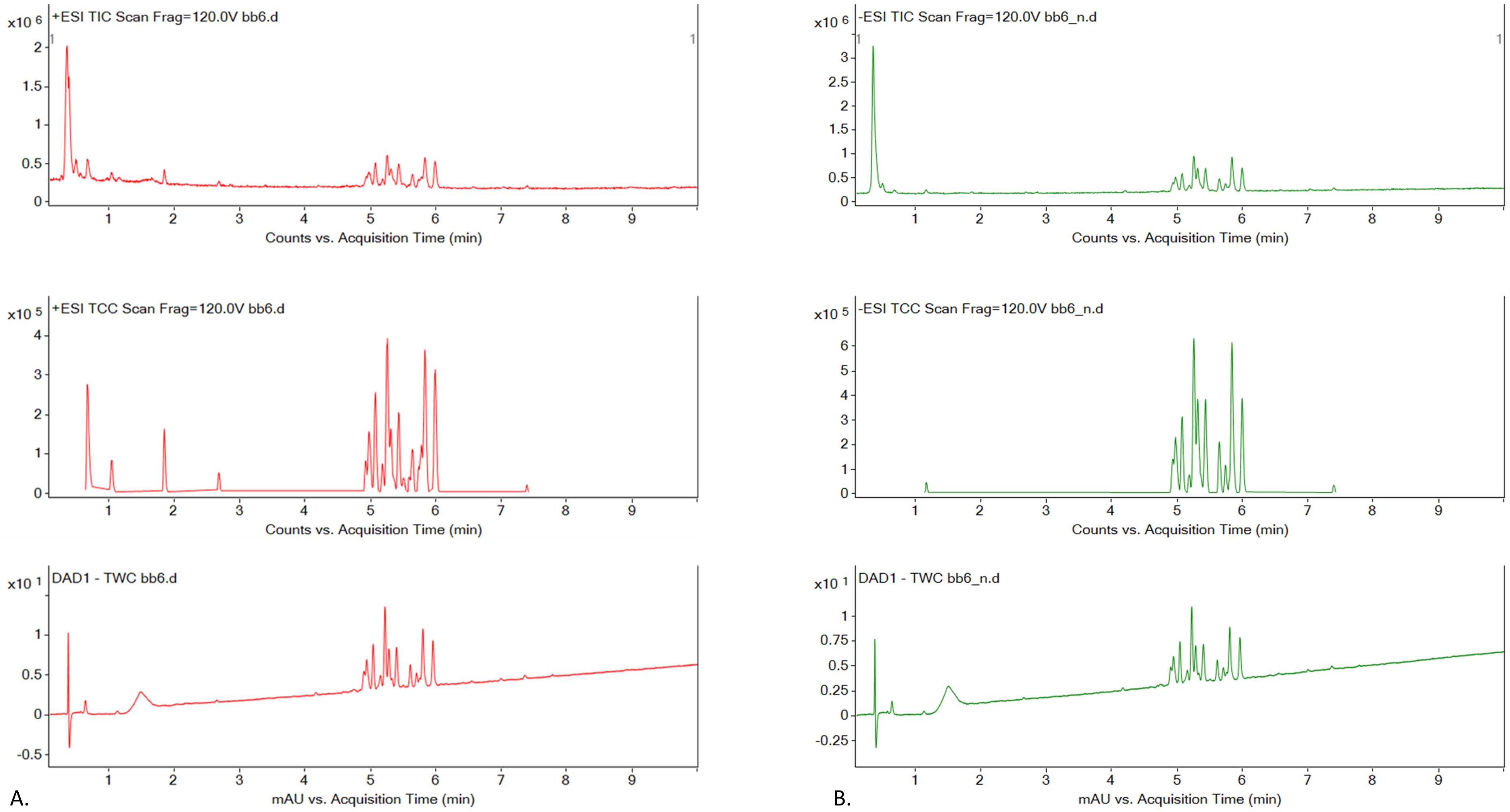

Figure S12. LC-ESI-MS of 6P-BCP-Bu extract in positive (A) and negative (B) ion modes.

S.3. GC-MS chromatograms of the fractions 1-BB-Et, 4-BB-Et and 6P-BCP-Et

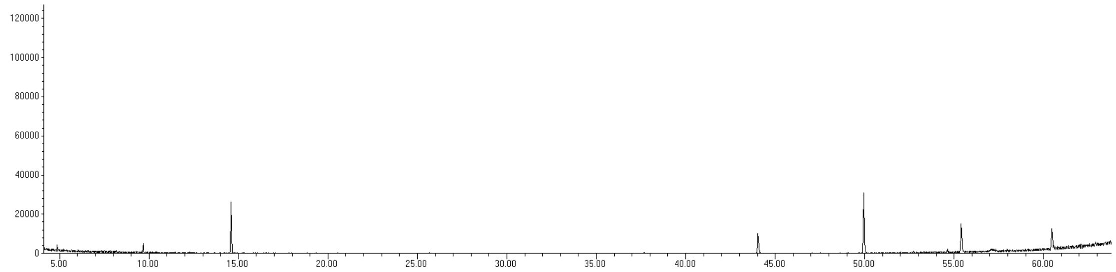

Figure S13. GC-MS chromatogram of 1-BB-Et.

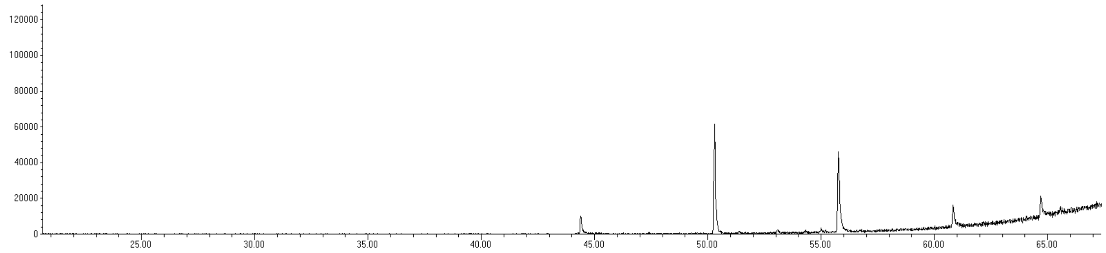

Figure S14. GC-MS chromatogram of 4-BB-Et.

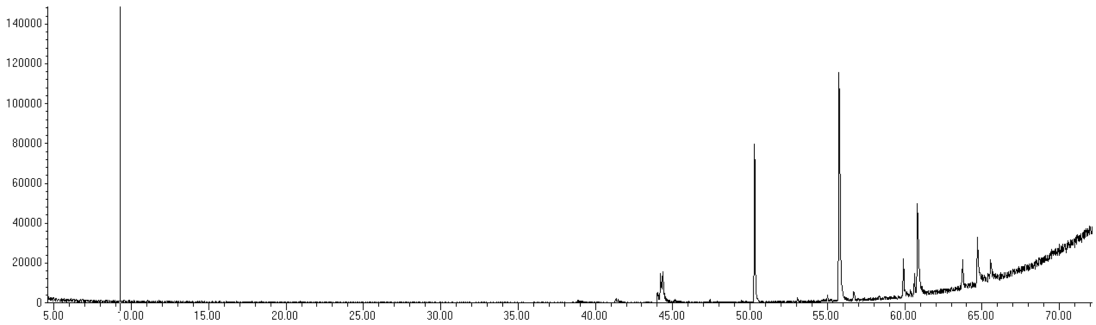

Figure S15. GC-MS chromatogram of 6P-BCP-Et.
